# The Role of Interleukin-28b Gene Polymorphisms in Chinese Patients With Chronic Hepatitis C Treated With Pegylated Interferon and Ribavirin

**DOI:** 10.5812/hepatmon.18793

**Published:** 2014-08-09

**Authors:** Yin Mi, Ying Tang Gao, Xiao Lei Jiao, Hua Guo, Tong Liu, Li Jing, Wen Xia Shi, Zhi Du

**Affiliations:** 1 Key Laboratory of Artificial Cell, Tianjin Third Central Hospital, Tianjin, China; 2 Radiotherapy Department, First Affiliated Hospital, Zhengzhou University, Henan, China

**Keywords:** China, Polymorphism, Hepatitis C, Interleukin-28B, Therapy

## Abstract

**Background:**

Interleukin-28B (IL28B) single nucleotide polymorphism (SNP) rs8099917 has been described to be associated with response to treatment with pegylated interferon and ribavirin (PEG-IFN/RBV) in patients with chronic hepatitis C from the North America, Europe, Asia countries like Japan and Taiwan. Whether this holds true for Chinese patients remains unknown.

**Objectives:**

We aimed to study the effects of IL28B rs8099917 on antiviral therapy responses in Chinese patients with hepatitis C.

**Patients and Methods:**

IL28B rs8099917 was genotyped in 263 patients with hepatitis C virus (HCV) infection and 244 healthy controls in Tianjin, China using TaqMan SNP genotyping method. The roles of rs8099917 and clinical characteristics in antiviral treatment were analyzed by logistic regression.

**Results:**

Among 263 patients with chronic HCV infection, 223 had a TT genotype (84.8%). Frequencies of TG/GG genotypes in patients with hepatitis C were significantly different from those of healthy controls (15.2% vs. 9.0%; P = 0.033). Patients with HCV infection had a higher G allele frequency than healthy controls (7.8% vs. 4.7%; P = 0.044). Univariate analysis revealed no significant association between rs8099917 and sustained virological response (SVR) (P = 0.612). However, it was found that HCV genotypes 2a/3a, age, prothrombin time (PT), albumin (ALB) and cholesterol (CHO) were associated with SVR. In multivariate analysis, only ALB was significantly an independent predictor of SVR (OR = 1.223; 95%CI: 1.046−1.430; P = 0.011).

**Conclusions:**

In contrast with T, rs8099917 G is a susceptible allele to HCV in China. ALB can independently predict SVR. Rs8099917 may play a quiet role to predict treatment response of patients with hepatitis C who received PEG−IFN/RBV therapy in China.

## 1. Background

HCV infected approximately 150 million people worldwide, resulting in death of up to 3.5 million people annually (1). Chronic HCV infection is a leading cause of liver cirrhosis and hepatocellular carcinoma (2). Current standard of care therapy for chronic HCV infection is PEG−IFN/RBV; however only 40−50% of patients infected with HCV genotype 1 achieve SVR. Moreover, the treatment is associated with significant side effects, which is tolerated in few patients (3-6).

IL28B is also called IFN−λ3 belonging to type III interferon family. The single nucleotide polymorphism (SNP) rs8099917 is located 8 kilobases upstream of the IL28B gene (7, 8). In recent years, three genome wide association studies identified the association between IL28B SNP rs8099917 and response to the treatment with PEG-IFN/RBV among Caucasian and Japanese patients infected with HCV (7-9). Rauch et al. (7) performed a research on 1362 Caucasian patients treated with PEG−IFN/RBV. G was regarded as a risk allele owing to strong association between rs8099917 G allele and treatment failure in their study (OR = 5.19; 95%CI: 2.90–9.30; P = 3.11×10^-8^). Suppiah et al. (8) documented that rs8099917 T allele could predict SVR (OR = 1.98; 95%CI: 1.57–2.52; P = 9.25×10^-9^). Furthermore, patients with TT genotype had a higher SVR rate compared to TG and GG genotypes. Afterwards several studies in cohorts of HCV genotype 1 patients in Japan, Taiwan, South Korea and Chile revealed that patients with TT genotype were more likely to obtain SVR than those carrying TG/GG genotypes (10-13). Paradoxically in the study undertaken by Chen et al. (14) in a Taiwanese cohort, GG genotype frequency of normal control males was 21.5 times more than that of male patients with HCV infection. GG could be considered as a protective genotype. In addition, Sinn et al. (15) studied Koreans with HCV genotype 1 infection and reported that there was no statistically difference in SVR rate between TT and TG/GG patients (P = 0.19). Therefore, it can be seen that the association between IL28B rs8099917 and response to treatment is not definite in Asian patients with chronic hepatitis C. The prevalence rate of hepatitis C is 1-1.9% in China (16) where seldom studies investigated the influence of rs8099917 on the treatment outcome of hepatitis C patients with PEG-IFN/RBV.

## 2. Objectives

In the study, we detected the genotype and allele frequencies of IL28B rs8099917 in a Chinese cohort of patients with HCV infection and healthy people. Then we analyzed the impact of IL28B rs8099917 gene polymorphisms on patients’ responses to therapy with PEG−IFN/RBV with an attempt to offer certain evidences to clinical workers for effective individualized treatment and early assessment of patients.

## 3. Patients and Methods

### 3.1. Patients

**Figure 1.**
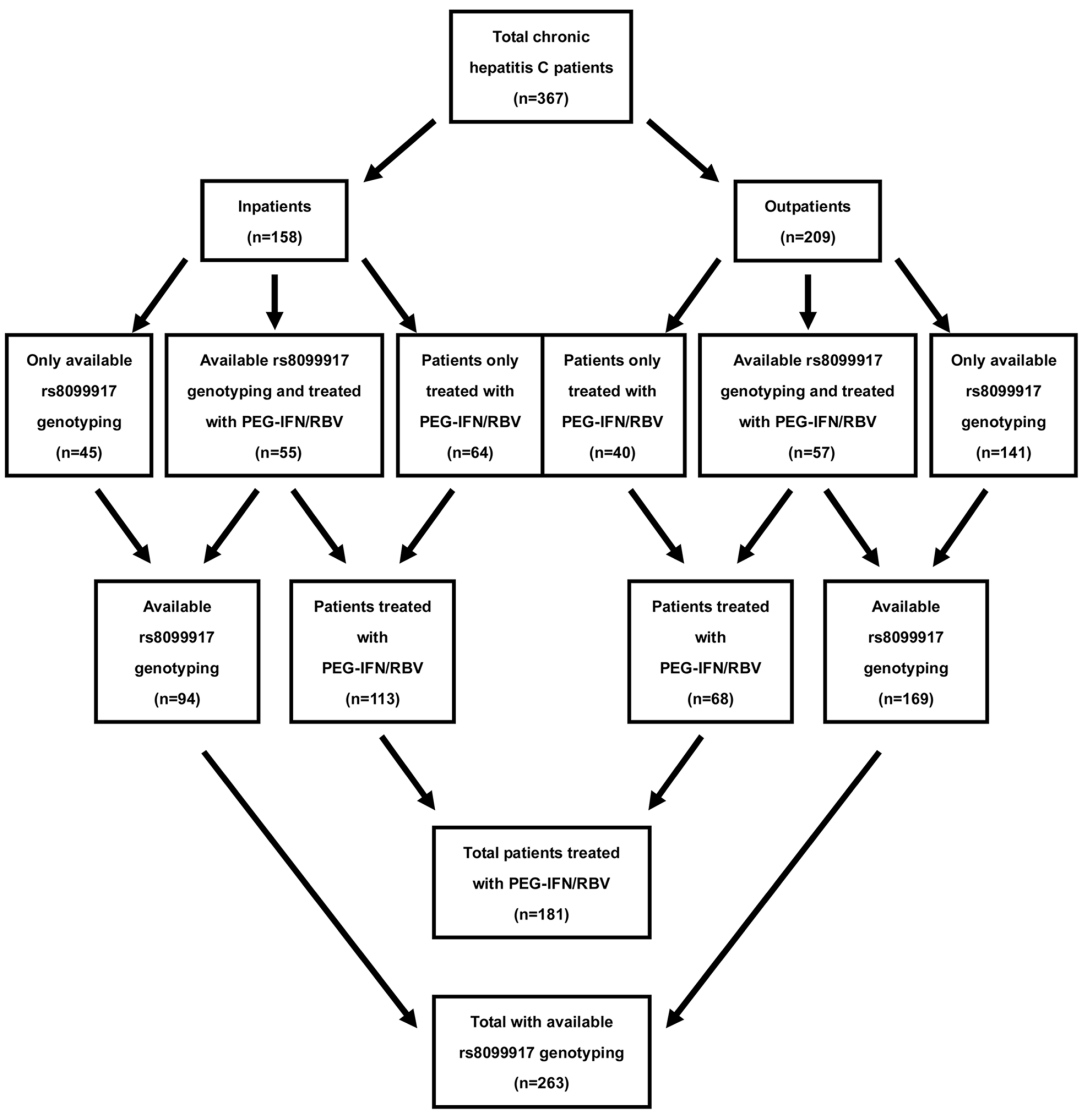
Overview of the Study Population Rs8099917 genotyping is available in 112 patients treated with PEG-IFN/RBV. HCV genotype was detected in 89 patients.

We retrospectively enrolled 367 patients with chronic infection of HCV from 2005 to 2010 at Tianjin Third Central Hospital, Tianjin, China. In total, 158 patients were hospitalized and 209 were outpatients. Because of compliance problem, we actually genotyped IL28B and HCV in 263 and 176 patients, respectively. In total, 181 patients were treated for 48 weeks with peg-IFN-α2a (180 μg/week; Shanghai Roche Co., LTD, Shanghai, China) subcutaneously and oral ribavirin (Shenyang Oasis Pharmaceutical Manufacturing Co., LTD, Shenyang, China) according to body weight (< 60 kg, 800 mg/day; 60-75 kg, 1000 mg/day; and > 75 kg, 1200 mg/day). The 181 patients’ serum HCV RNA load results before treatment, after 12 weeks of treatment, at the end of treatment and at 24 weeks after completing therapy were documented in clinical records. IL28B rs8099917 was genotyped in 112 of 181 patients and HCV genotypes were detected in 89 of 112 patients (Figure 1). Chronic hepatitis C was diagnosed in all patients included in the study according to the diagnostic criteria of hepatitis C (3). The exclusion criteria were as follows: human immunodeficiency virus infection, autoimmune hepatitis, a psychiatric condition and previous liver transplantation. Clinical data of each inpatient was obtained from their medical records by treating physicians. In addition, 244 healthy people who underwent health examination in the department of health of Tianjin Third Central Hospital were selected as healthy controls. This study was approved by the Ethical Committee of Tianjin Third Central Hospital, and informed consent was obtained from each patient before the study.

### 3.2. Definition of Response

For the analysis of response to standard of care PEG-FN/RBV, SVR was defined as undetectable HCV RNA at the end of treatment and 24 weeks after the treatment completion. Relapse was defined as undetectable levels of HCV RNA at the end of treatment, while detectable HCV-RNA levels at 24 weeks after cessation of treatment. Patients whose HCV-RNA levels remained detectable at the end of treatment were considered to be nonresponders. Relapse and nonresponse composed treatment failure.

### 3.3. HCV RNA Detection and HCV Genotyping

Serum HCV RNA was measured using diagnostic kit for quantification of hepatitis C virus RNA (Shanghai Huake Bio-engineering Co., LTD, Shanghai, China) according to the manufacturer’s protocol with a detection limit of 500 IU/mL. HCV genotyping was performed with the oligonucleotide DNA chip by the combined reverse transcription-asymmetric polymerase chain reaction (PCR) and cohybridization methods (17).

### 3.4. Genomic DNA Extracting

Genomic DNA was obtained from peripheral blood white blood cells of patients and healthy controls with phenol-chloroform-isoamyl alcohol extraction method, and extraction results were detected in 1.5% agarose gel electrophoresis.

### 3.5. IL28B rs8099917 Genotyping

For 263 patients whose DNA was available, rs8099917 genotyping was performed by PCR amplification with ABI PRISM ® 7000 SDS (Applied Biosystems, Foster City, CA, USA). The PCR amplification mixture was 10 µL in a total volume containing 1.5 µL DNA template, 5 µL 2 × TaqMan Genotyping Master Mix, 0.25 µL 40 × TaqMan SNP Genotyping Assay Mix (rs8099917, C_11710096_10), and 3.25 µL ultrapure water. The PCR reactions were performed in 96-well microplates with three negative control (NTC). Reaction conditions were: 50 cycles of thermal denaturation at 95 ℃ for 5 minutes, denaturation at 92ºC for 20 seconds and annealing at 60ºC for 1 minute. Two allelic-specific TaqMan probes were used to detect a specific SNP target. Allele discrimination was achieved by the detection of fluorescent signal and analysis of the distribution of the scatter plot chart using ABI PRISM ® 7000 SDS (Applied Biosystems, Foster City, CA, USA).

### 3.6. Statistical Analysis

The Hardy-Weinberg disequilibrium test was performed for SNP rs8099917 of patients group with chronic hepatitis C and healthy control group. The logarithmic transformation of the original values was performed before measuring serum HCV RNA levels. Comparisons between continuous variables in the two groups were performed using the Mann-Whitney U test or the Student T-test. Categorical data were compared using the χ^2^ test, correction for continuity or Fisher’s exact test. Logistic regression analysis was performed to determine SVR predictors. The odds ratios (OR) and 95% confidence intervals (95% CI) were calculated. Data was analyzed by SPSS 17.0 statistical software package (SPSS Inc., Chicago, USA). All statistical analyses were based on the two-sided hypothesis tests, and P values < 0.05 were considered statistically significant.

## 4. Results

### 4.1. Basic Demographic Characteristics of Patients With Chronic HCV Infection 

Basic demographic characteristics of patients are presented in Table 1 after consultation of clinical records and statistical disposal. There were 185 males (53.2%). In total, HCV genotype was detected in 176 patients, with 132 (75.0%) infected with HCV genotype 1b, 43 (24.4%) with HCV genotype 2a and 1 (0.6%) with 3a. The mean age was 56.7 ± 12.8 years. Among 158 inpatients, 96.8% were Chinese Han, and the mean of prothrombin time (PT), albumin (ALB) and cholesterol (CHO) were 15.9 ± 2.62 seconds, 33.6 ± 5.84 g/L and 3.32 ± 1.06 mmol/L, respectively. Liver cirrhosis was diagnosed in 88% of inpatients by clinical and radiographic data. Some medical records were not available due to retrospective nature of the study. Of 181 patients receiving PEG−IFN/RBV therapy, 63 obtained SVR, 73 were nonresponders and 45 relapsed. The overall SVR rate was 34.8% regardless of HCV genotype. 

**Table 1.** Basic Demographic Characteristics of Patients With Chronic HCV Infection ^a,b^

Clinical characteristics	No.	value
**Age, y, mean ± SD**	320	56.7 ± 12.8
**Male, n/No. (%)**		185/348 (53.2)
**Han population, n/No. (%)**		153/158 (96.8)
**HbsAg +, n/No. (%)**		14/117 (12.0)
**GGT-Ⅱ +, n/No. (%)**		23/125 (18.4)
**Smoking history, n/No. (%)**		47/151 (31.1)
**Drinking history, n/No. (%)**		39/153 (25.5)
**Blood transfusion history, n/No. (%)**		28/158 (17.7)
**Liver cirrhosis**		139/158 (88.0)
**HCV 1b, n/No. (%)**		132/176 (75.0)
**HCV-RNA > 400 000IU/mL, n/No. (%)**		180/292 (61.6)
**HCV-RNA, log IU/mL, median (IQR)**	292	5.97 (5.18-6.56)
**ALT, U/L, median (IQR)**	158	55 (31.8-96.5)
**AST, U/L, median (IQR)**	155	61 (42-97)
**DBIL, umol/L, median (IQR)**	158	8.6 (5.4-14.0)
**TBIL, umol/L, median (IQR)**	158	22.4 (13.7-36.1)
**AFP, ng/mL, median (IQR)**	134	15.9 (8.3-49.0)
**AFU, U/L, median (IQR)**	128	464.5 (36.3-637.3)
**PT (s), mean ± SD**	158	15.9 ± 2.62
**PLT, ×109/L, mean ± SD**	158	98.5 ± 69.7
**ALP, U/L, mean ± SD**	156	91.0 ± 41.3
**ALB, g/L, mean ± SD**	157	33.6 ± 5.84
**CHO, mmol/L, mean ± SD**	115	3.32 ± 1.06
**TG, mmol/L, median (IQR)**	115	0.93 (0.70-1.39)

^a^ All data were obtained only from 158 inpatients except age, gender, HCV genotype and HCV virus load.

^b^ Abbreviations: HbsAg, hepatitis B virus surface antigen; GGT-Ⅱ, γ-glutamyl transferase isoenzymesⅡ; ALT, alanine aminotransferase; AST, aspartate aminotransferase; DBIL, direct bilirubin; TBIL, total bilirubin; AFP, alpha fetoprotein; AFU, α-L-fucosidase; PT, prothrombin time; PLT, platelet; ALP, alkaline phosphatase; ALB, albumin; CHO, cholesterol; TG, triglyceride; IQR, Intra-quartile range (25th percentile–75th percentile); SD, standard deviation.

### 4.2. Comparison of rs8099917 Genotypes and Alleles Between Patients and Healthy Controls

Both genotypes distribution of patients and healthy controls were in Hardy-Weinberg equilibrium (χ^2^ = 0.26; χ^2^ = 0.43) and both P values were more than 0.05. Of 263 patients whose rs8099917 were genotyped, 223 (84.8%) had the TT genotype, 39 (14.8%) had the TG genotype, and 1 (0.40%) carried GG genotype. The frequency of the rs8099917 T allele was 0.922. Among 244 healthy controls, the number of people who carried TT, TG and GG genotypes were 222 (91.0%), 21 (8.6%) and 1 (0.4%), respectively. The T allele frequency was 0.953 (Table 2). TG/GG rate in patients with chronic hepatitis C was higher than that in healthy controls (15.2% vs. 9.0%; OR = 1.810; 95%CI: 1.042–3.145; P = 0.033). Patients with chronic hepatitis C had a significantly higher frequency of G allele compared with healthy controls (7.8% vs. 4.7%; OR = 1.709; 95%CI: 1.010–2.893; P = 0.044). By comparison with T, population with rs8099917 G allele is susceptible to HCV. Therefore, rs8099917 G was considered as a susceptible allele to HCV compared with T.

**Table 2.** Comparison of Rs8099917 Genotype and Allele Distribution Between Patients and Healthy Controls ^a,b^

	Healthy Controls, N = 244	Patients, N = 263	OR (95%CI)	P Value
**Genotype**				0.033 ^c^
TT	222 (91.0)	223 (84.8)	1	
TG	21 (8.6)	39 (14.8)		
GG	1 (0.4)	1 (0.4)		
TG/GG	22 (9.0)	40 (15.2)	1.810 (1.042-3.145)	
**Allele**				0.044 ^c^
T	465 (95.3)	485 (92.2)	1	
G	23 (4.7)	41 (7.8)	1.709 (1.010-2.893)	
Hardy-Weinberg, χ^2^	0.43	0.26		
Hardy-Weinberg, P	0.5 ＜ P ＜ 0.75	0.5 ＜ P ＜ 0.75		

^a^ Data are presented as No. (%).

^b^ Abbreviations: OR, odds ratio; CI, confidence interval.

^c^ Pearson χ^2^ test.

### 4.3. Association of IL28B rs8099917 Gene Polymorphisms and Treatment Outcome

Regardless of HCV genotype, 112 patients with PEG−IFN/RBV therapy whose rs8099917 were genotyped were included in this analysis. Among them, 97 had TT genotype and 15 carried TG/GG genotypes. The SVR rate in patients with TT genotype was higher than those with TG/GG genotypes; although, the difference was not statistically significant (40.2% vs. 33.3%; OR = 1.345; 95%CI: 0.427–4.237; P = 0.612, Table 3). The SVR rates for T and G alleles were 41.6% and 31.3%, respectively. No significant difference was found likewise (OR = 1.566; 95%CI: 0.526–4.666; P = 0.417, Table 3). Meanwhile, 89 of the 112 patients were genotyped for HCV. Of 72 patients with HCV genotype 1b infection, no association was found between rs8099917 gene polymorphisms and treatment outcome (P = 1.000, Table 3). The association between rs8099917 and treatment outcome was not analyzed solely among patients with HCV genotypes 2a/3a infection due to the small sample size.

**Table 3.** Influence of IL-28B Rs8099917 Gene Polymorphisms on Treatment Outcome ^a^

	TT, No. (%)	TG/GG, No. (%)	OR (95%CI)	P value	T, No. (%)	G, No. (%)	OR (95%CI)	P Value
**Total, N = 112**								0.417 ^b^
Treatment Failure	58 (59.8)	10 (66.7)	1		125 (58.4)	11 (68.8)	1	
SVR	39 (40.2)	5 (33.3)	1.345 (0.427-4.237)	0.612 ^b^	89 (41.6)	5 (31.3)	1.566 (0.526-4.666)	
**HCV 1 ** ^**c**^ **, N = 72 ^c^**								1.000 ^d^
Treatment Failure	49 (79)	8 (80)	1		105 (78.9)	9 (81.8)	1	
SVR	13 (21)	2 (20)	1.061 (0.201-5.614)	1.000 ^d^	28 (21.1)	2 (18.2)	1.200 (0.245-5.872)	

^a^ Abbreviations: SVR, sustained virological response; OR, odds ratio; CI, confidence interval.

^b^ Pearson χ2 test

^c^ Rs8099917 genotyping was not available in 3 of 75 patients infected with HCV genotype 1b

^d^ Correction for continuity

### 4.4. The Role of rs8099917 and Clinical Characteristics to Predict SVR

The impacts of rs8099917 and clinical characteristics on SVR were evaluated by logistic regression (Table 4). It was found that there was no significant association between rs8099917 and SVR (P = 0.612) by univariate analysis. However, five variables associated with SVR were HCV genotypes 2a/3a infection (OR = 3.556; 95%CI: 1.174–10.765; P = 0.044), age (OR = 0.958; 95%CI: 0.929–0.987; P = 0.005), PT (OR = 0.780; 95%CI: 0.631–0.965; P = 0.022), ALB (OR = 1.188; 95%CI: 1.086–1.300; P = 0.000) and CHO (OR = 2.078; 95%CI: 1.224–3.531; P = 0.007). High SVR rate was more probable in patients with HCV genotypes 2a/3a infection, younger age, shorter PT, higher lever of ALB or CHO. Compared to 15 of 75 patients infected with HCV genotype 1b, 8 of 17 patients infected with HCV genotypes 2a/3a obtained SVR (SVR rate 20.0% vs. 47.1%; P = 0.044). After excluding HCV genotype because of its many missing values, we assessed the relative contribution of important variables to treatment response in multivariate analysis and confirmed only ALB as an independent predictor of SVR (OR = 1.223; 95%CI: 1.046–1.430; P = 0.011, Table 4).

**Table 4.** Logistic Regression Analysis of Factors Associated with SVR ^a^

Variable	No.	Univariate analysis, OR (95%CI)	P Value	Multivariate analysis ^b^, OR (95%CI)	P Value
**Gender**	178				
Female		1			
Male		1.115 (0.597-2.082)	0.733		
**Smoking history**	106				
No		1			
Yes		0.894 (0.346-2.311)	0.818		
**Drinking history**	108				
No		1			
Yes		0.775 (0.291-2.063)	0.609		
**GGT-Ⅱ**	86				
−		1			
+		0.816 (0.261-2.556)	0.727		
**HCV genotype**	92				
1b		1			
2a/3a		3.556 (1.174-10.765)	0.044	-	-
**HCV-RNA virus load**	160				
HCV-RNA > 400 000IU/mL		1			
HCV-RNA < 400 000IU/mL		1.044 (0.513-2.124)	0.906		
**rs8099917**	112				
TG/GG		1			
TT		1.345 (0.427-4.237)	0.612		
**HCV-RNA, log IU/mL**	160	1.000 (1.000-1.000)	0.420		
**Age, y**	171	0.958 (0.929-0.987)	0.005	0.997 (0.942-1.056)	0.923
**ALT, U/L**	113	1.002 (0.999-1.005)	0.188		
**AST, U/L**	110	1.000 (0.996-1.004)	0.993		
**DBIL, umol/L**	113	0.959 (0.911-1.010)	0.115		
**TBIL, umol/L**	113	0.975 (0.948-1.002)	0.072		
**AFP, ng/mL**	95	1.000 (0.999-1.001)	0.567		
**AFU, U/L**	89	1.001 (0.999-1.002)	0.373		
**PT, s**	113	0.780 (0.631-0.965)	0.022	0.967 (0.643-1.456)	0.874
**PLT, × 10** ^**9**^ **/L**	113	1.004 (0.998-1.010)	0.162		
**ALP, U/L**	112	1.006 (0.996-1.016)	0.213		
**ALB, g/L**	112	1.188 (1.086-1.300)	0.000	1.223 (1.046-1.430)	0.011
**CHO, mmol/L**	82	2.078 (1.224-3.531)	0.007	1.424 (0.753-2.694)	0.277
**TG, mmol/L**	82	1.300 (0.907-1.863)	0.153		

^a^ Abbreviations: GGT-Ⅱ, γ-glutamyl transferase isoenzymes Ⅱ; ALT, alanine aminotransferase; AST, aspartate aminotransferase; DBIL, direct bilirubin; TBIL, total bilirubin; AFP, alpha fetoprotein; AFU, α-L-fucosidase; PT, prothrombin time; PLT, platelet; ALP, alkaline phosphatase; ALB, albumin; CHO, cholesterol; TG, triglyceride; OR, odds ratio; CI, confidence interval.

^b^ Multivariate analysis was performed with factors significantly associated with SVR by univariate analysis except for HCV genotype because of its many missing values in multivariate analysis. Finally, multivariate analysis was performed in 82 patients.

## 5. Discussion

Not every patient with hepatitis C infection receiving standard of care PEG-IFN/RBV could achieve SVR. Therefore, the treatment not only is costly but also has many side effects, which few patients could tolerate. If the predictors of treatment outcome be identified, the situation would be greatly improved. In addition to the viral and treatment factors, studies have found that IL28B gene polymorphism is closely related with the treatment response in hepatitis C infection patients treated with PEG-IFN/RBV in the recent years. SNP rs8099917 and rs12979860 have a higher association with treatment response in comparison with other SNPs located in IL28B gene and are focused on in the recent years. Unfortunately, the association is diverse in different races and HCV genotypes in current studies. In this study, we detected the genotype and allele frequencies of IL28B rs8099917 in Chinese patients with HCV infection and healthy people and assessed the influence of IL28B rs8099917 gene polymorphisms on treatment responses in patients with chronic hepatitis C treated with PEG-IFN/RBV.

So far, there have been few studies investigating the association between IL28B rs8099917 gene polymorphisms and treatment response to therapy with PEG-IFN/RBV in Chinese patients with chronic hepatitis C. In our study, frequencies of T allele in healthy people and patients chronically infected with HCV were 95.3% and 92.2%, respectively. The frequencies of TT genotype were 91.0% and 84.8%, respectively. Healthy people have higher frequencies of T allele and TT genotype compared with patients with HCV infection (P = 0.044; P = 0.033). Therefore, TT genotype was presumed to be protective. Distributions of rs8099917 genotypes and alleles in our study are similar to those previously reported in other Asian countries and regions. As some studies reported, TT genotype frequencies of healthy people in Taiwan and Japan were 89.6% and 83.7%. While they were 89.6%, 86.2% and 70.4% for patients with HCV infection in Taiwan, Korea and Japan, respectively. Healthy people had a higher frequency of TT genotype than patients with chronic hepatitis C in Japan. Dissimilarly, there was no obvious difference in the frequency of TT genotype between Taiwanese patients with hepatitis C and healthy people, and TT genotype frequency of Japanese patients with chronic hepatitis C was lower than that of patients in other Asian countries and regions (10, 11, 14, 18). However, in European and Argentine patients infected with HCV, the frequency of TT genotype were 58.0% and 40.0% (7, 19), respectively, which were both lower than those of Asian patients. This may to some extent explain why Asian patients with hepatitis C receiving PEG-IFN/RBV treatment have a higher SVR rate than Caucasian ones (20).

In recent years, studies from different ethnic groups revealed that IL28B rs8099917 gene polymorphism was associated with treatment response to PEG−IFN/RBV in patients with HCV infection (7, 9, 11, 12, 19). Suppiah et al. (8) evaluated 848 Caucasian patients with HCV genotype 1 infection and found that rs8099917 was related with SVR (OR = 1.98; 95%CI: 1.57-2.52; P = 9.25 × 10^−9^). Among patients achieving SVR, 63% had TT genotype while 37% carried TG/GG genotypes. Tanaka et al. (9) investigated 142 patients infected with HCV genotype 1 within a Japanese population. The results demonstrated that rs8099917 was strongly associated with both SVR and null virological response (P = 1.11 × 10^−27^; P = 3.11 × 10^−15^). However, results of our research in Chinese patients with chronic HCV infection were not completely consistent with those previously reported. In our study though the SVR rate was higher in TT patients than TG/GG ones (40.2% vs. 33.3%), the difference was not statistically significant (P = 0.612). Rs8099917 gene polymorphism was not associated with SVR in patients with HCV genotype 1b infection (P = 1.000). Logistic regression showed that rs8099917 was not a predictor of SVR. Being similar to our results, Sinn et al. (15) reported that SVR rate did not differ significantly in HCV genotype 1 infection patients with TT genotype and TG/GG genotypes in Korea (P = 0.19). Therefore, it is presumed that due to the high frequency of protective TT genotype and the low frequency of risk GG genotype, the IL28B gene polymorphism may play a quiet role in predicting treatment response of Chinese hepatitis C patients treated with PEG-IFN/RBV (15). On the other hand, the difference may be resulted from the small sample size in our study.

In addition, it was found by univariate analysis that HCV genotypes 2a/3a infection, younger age, shorter PT, higher level of ALB and CHO were associated with higher SVR rates. The findings that HCV genotypes 2a/3a, age and CHO are associated with SVR are in accordance with previous studies. Idrees et al. evaluated 400 patients with chronic hepatitis C (21). The result showed that patients with HCV genotypes 2a/3a infection or less than 40 years old had higher SVR rates (P = 0.022; P = 0.005). In the study of Weich et al. (22), patients with HCV genotype 1 infection with a younger age or higher level of CHO were more likely to obtain SVR. Unlike previous studies which failed to consider the impact of PT and ALB on predicting treatment outcome, our study showed PT and ALB as predictors of SVR. These predictors of SVR may assist clinical physicians in early evaluating the progress of disease and patients prognosis.

SVR rates in our study were much lower than those previously reported. Standard of care PEG−IFN/RBV in this study leads to a SVR in 34.8%, 20.0% and 47.1% of total patients, patients infected with HCV genotype 1b and HCV genotypes 2a/3a. A previous study showed that SVR was achieved in 42-52% of patients with HCV genotype 1 infection and 76-82% of those with HCV genotypes 2/3 infection (23). The SVR rates in other Asian countries and regions (Japan, Taiwan, South Korea) were also higher than those in this study (10, 14, 15). The following reasons may explain this problem. First of all, cirrhosis was a difficult to treat characteristic able to reduce SVR rate (24). Idrees et al. (21) found that none of 16 patients with cirrhosis achieved SVR in their study. In this study, patients with liver cirrhosis account for 88% of inpatients, which may lead to a low SVR rate. Although Ochi et al. (10) found higher SVR rates in Japanese and Taiwanese patients, patients in their study were in fibrosis stage not showing cirrhosis. Secondly, not all the outpatients selected in this study were treated initially. However, the specific number was not put on record. Retreatment of nonresponders usually produced low SVR rates ranging from 7% to 18% (24). Furthermore, small sample size may be another factor. Beside all the factors above, the fact that HCV genotype 1b was predominant in this study (75%) was associated with a decreased overall SVR to treatment.

In summary, this study analyzed the distribution feature of IL28B rs8099917 in a Chinese cohort and the effect of rs8099917 on treatment response to PEG-IFN/RBV in Chinese patients with HCV infection. Besides, supplemented the multiple ethnic studies of association between IL28B rs8099917 and treatment outcome of patients with chronic hepatitis C receiving PEG-IFN/RBV therapy and confirmed several predictors of treatment outcome to offer certain evidences to clinical practitioners for effective individualized treatment and early evaluation of patients. A major limitation of our study was the small number of patients with available rs8099917 genotyping and PEG-IFN/RBV therapy because of its retrospective nature. In future, there is a need for multicenter studies with larger sample size to identify the specific role of IL28B in antiviral treatment of patients with hepatitis C.

## Supporting Information

Supplement 1. IL28B Rs8099917 Genotyping (A) PCR amplification curve of IL28B rs8099917. (B) Analysis graph of IL28B rs8099917 polymorphisms. Part of rs8099917 genotyping results are shown in this graph. Two allelic-specific TaqMan probes (VIC and FAM) were used to detect a specific SNP target. Rhombus, round and triangle indicate genotype TT, GG, and TG, respectively. Square represents negative control (NTC).

## Appendix

**Appendix 1.** The Association between IL-28B Rs8099917 Genotypes and Clinical Features of Patients with HCV Infection ^a^

Variable	No.	rs8099917, TT	rs8099917, TG/GG	P Value
**Gender, No. (%)**	247			0.193 ^b^
Male		110 (51.9)	14 (40.0)	
Female		102 (48.1)	21 (60.0)	
**HbsAg, No. (%)**	71			1.000 ^c^
+		7 (11.1)	0 (0)	
–		56 (88.9)	8 (100)	
**GGT-Ⅱ, No. (%)**	78			0.586 ^c^
+		9 (12.9)	0 (0)	
–		61 (87.1)	8 (100)	
**Blood transfusion history, No. (%)**	94			0.724 ^d^
Yes		17 (20.2)	1 (10.0)	
No		67 (79.8)	9 (90.0)	
**HCV genotype, No. (%)**	149			0.230 ^d^
1b		96 (73.8)	17 (89.5)	
2a/3a		34 (26.2)	2 (10.5)	
**HCV-RNA virus load, No. (%)**	211			0.395 ^b^
HCV-RNA > 400 000IU/mL		106 (59.9)	23 (67.6)	
HCV-RNA < 400 000IU/mL		71 (40.1)	11 (32.4)	
HCV-RNA, log IU/mL; median (IQR)	211	5.95 (5.19-6.61)	5.95 (5.42-6.35)	0.610 ^d^
**Age, y; mean ± SD**	227	55.6 (12.4)	55.8 (11.7)	0.870 ^e^
**ALT, U/L; median (IQR)**	94	50 (25.8-79.0)	44 (34.8-69.5)	0.878 ^e^
**AST, U/L; median (IQR)**	92	57 (34-95)	60 (28.0-85.5)	0.655^e^
**DBIL, umol/L; median (IQR))**	94	8.3 (5.1-12.5)	5.6 (4.4-17.8)	0.544 ^e^
**TBIL, umol/L; median (IQR)**	94	19.6 (13.6-31.4)	15.5 (11.2-27.8)	0.333 ^e^
**AFP, ng/mL; median (IQR)**	80	15.3 (8.2-32.0)	13.9 (11.1-113.6)	0.386 ^e^
**AFU, U/L; median (IQR)**	79	282 (24-538)	45.6 (32-443)	0.603 ^e^
**PT, s; mean ± SD**	94	15.8 (2.45)	14.8 (1.23)	0.199 ^f^
**PLT, ×10** ^**9**^ **/L; mean ± SD**	94	100 (63.7)	95.7 (55.5)	0.835^f^
**ALP, U/L; mean ± SD**	92	85.5 (37.4)	93.9 (36.2)	0.523 ^f^
**ALB, g/L; mean ± SD**	93	33.8 (6.09)	37.1 (5.17)	0.109 ^f^
**CHO, mmol/L; mean ± SD**	67	3.39 (1.09)	3.71 (1.02)	0.465 ^f^
**TG, mmol/L; mean ± SD**	67	1.13 (0.70)	1.29 (0.58)	0.300 ^e^

^a^ Abbreviations: HbsAg, hepatitis B virus surface antigen; GGT-Ⅱ, γ-glutamyl transferase isoenzymes Ⅱ; ALT, alanine aminotransferase; AST, aspartate aminotransferase; DBIL, direct bilirubin; TBIL, total bilirubin; AFP, alpha fetoprotein; AFU, α-L-fucosidase; PT, prothrombin time; PLT, platelet; ALP, alkaline phosphatase; ALB, albumin; CHO, cholesterol; TG, triglyceride; IQR, Intra-quartile range (25th percentile–75th percentile); SD, standard deviation.

^b^ Pearson χ^2^ test.

^c^ Fisher’s exact test.

^d^ Correction for continuity.

^e^ Mann-Whitney U test.

^f^Student t test.
